# Rapid classification of micro-particles using multi-angle dynamic light scatting and machine learning approach

**DOI:** 10.3389/fbioe.2022.1097363

**Published:** 2022-12-16

**Authors:** Xu He, Chao Wang, Yichuan Wang, Junxiao Yu, Yanfeng Zhao, Jianqing Li, Mubashir Hussain, Bin Liu

**Affiliations:** ^1^ Jiangsu Province Engineering Research Center of Smart Wearable and Rehabilitation Devices, School of Biomedical Engineering and Informatics, Nanjing Medical University, Nanjing, China; ^2^ The State Key Laboratory of Bioelectronics, School of Instrument Science and Engineering, Southeast University, Nanjing, China; ^3^ Changzhou Medical Center, The Affiliated Changzhou Second People’s Hospital of Nanjing Medical University, Changzhou Second People’s Hospital, Nanjing Medical University, Changzhou, China

**Keywords:** micro-particles detection, dynamic light scattering, MIE scattering, machine learning, features selection, shapley value

## Abstract

The rapid classification of micro-particles has a vast range of applications in biomedical sciences and technology. In the given study, a prototype has been developed for the rapid detection of particle size using multi-angle dynamic light scattering and a machine learning approach by applying a support vector machine. The device consisted of three major parts: a laser light, an assembly of twelve sensors, and a data acquisition system. The laser light with a wavelength of 660 nm was directed towards the prepared sample. The twelve different photosensors were arranged symmetrically surrounding the testing sample to acquire the scattered light. The position of the photosensor was based on the Mie scattering theory to detect the maximum light scattering. In this study, three different spherical microparticles with sizes of 1, 2, and 4 μm were analyzed for the classification. The real-time light scattering signals were collected from each sample for 30 min. The power spectrum feature was evaluated from the acquired waveforms, and then recursive feature elimination was utilized to filter the features with the highest correlation. The machine learning classifiers were trained using the features with optimum conditions and the classification accuracies were evaluated. The results showed higher classification accuracies of 94.41%, 94.20%, and 96.12% for the particle sizes of 1, 2, and 4 μm, respectively. The given method depicted an overall classification accuracy of 95.38%. The acquired results showed that the developed system can detect microparticles within the range of 1–4 μm, with detection limit of 0.025 mg/ml. Therefore, the current study validated the performance of the device, and the given technique can be further applied in clinical applications for the detection of microbial particles.

## 1 Introduction

Microparticles are small spherical particles with different size ranges within 1–1,000 μm (Li et al., 2013; Veremchuk et al., 2021). In the atmosphere, particles with a diameter less than 2.5 μm have the characteristics of strong activity, long residence time, and transport distance, which significantly harms human health and the quality of the atmosphere. The application of rapid classification of microparticles is to prevent damage to humans from airborne pollution and food contamination (Wieland et al., 2022). Particles can also be used as an effective drug delivery transmitter in cancer treatment and prevention (Nakane, 2012; Gong et al., 2015; Kumar et al., 2015). Light obscuration test (LOT) is an analytical method in which particles in a liquid are placed between a laser light source and a detector. A laser light source is used to illuminate the particles, thus creating a blocking light. The system processes the detected signal to display a calibration curve. The calibration curve quantifies the particles and determines their size (Hawe et al., 2013). Microparticle detection instruments commonly use LOT to detect insoluble particles in intravenous fluids for drug detection. The rapid classification of particles with different sizes is crucial for the human environment and the timely identification of microorganisms (Kumar et al., 2015). In the last decade, many microparticle detection techniques have been developed (Zhao et al., 2011; Dalili et al., 2019; Lengyel et al., 2019; Lerche, 2019), such as flow microscopy, spectroscopy, mass spectrometry (Zwicker, 2010; McNay et al., 2011; Kreimer et al., 2015). Recently, classifying particles using high-speed microscopy to acquire particle images by artificial intelligence algorithms has become a mainstream research method. Luo et al. (2020) developed a pipeline based on machine learning to identify images obtained from Charge Coupled Device (CCD) imaging and improved the accuracy of particle identification. Lugnan et al. (2020) developed a machine learning method for high-throughput single particle analysis using flow cytometry to achieve interference pattern classification of transparent PMMA microparticles with diameters of 15.2 and 18.6 μm. Bals et al. (2022) used scanning electron microscopy images to record contrast ratio and resolution, then classified the acquired images by machine learning based on the shape and size of micro-particles. The above methods are limited to imaging analysis and require more space for expensive detection instruments (Klug et al., 2019; Di et al., 2022; Yue et al., 2022).

**FIGURE 1:**
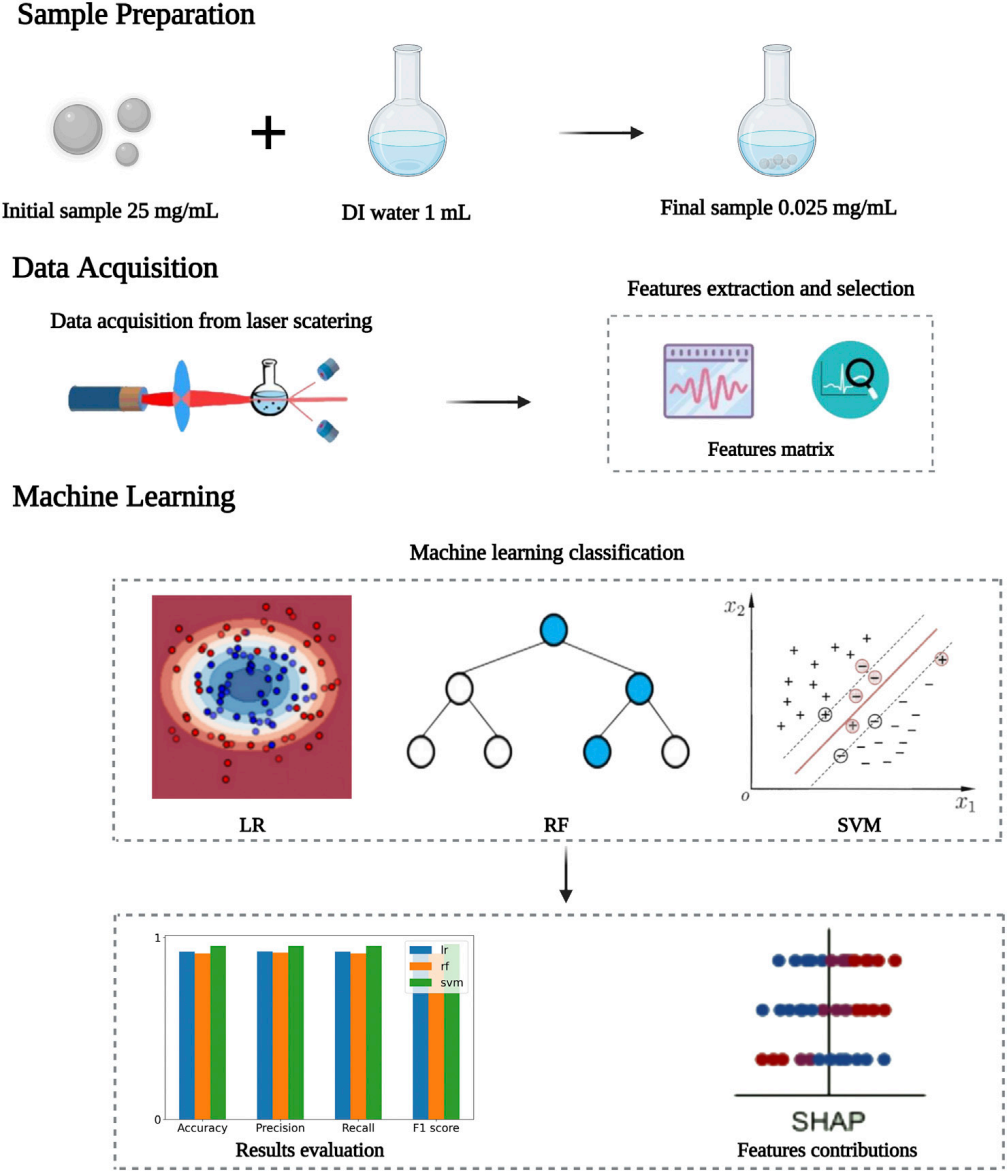
The flowchart for the detection principle for classifying microparticles based on MDLS and machine learning.

In this study, we proposed an application of multi-angle dynamic light scattering (MDLS) method based on machine learning. The initial sample was diluted by mixing 1 μl of the original sample (25 mg/ml) with deionized water (DI). The prepared sample was placed in the device to collect scattered light for 30 min. The surrounding photodetector acquired the multi-angle dynamic light scattering signal and converted the acquired scattered light signal into a voltage wave. The power spectrum features were obtained from the signal waveform, and the principal component analysis (PCA) and recursive feature elimination (RFE) methods were applied to select the optimum features. Machine learning (ML) is an artificial intelligence technique that enables fast and automated classification of input features. ML has been widely applied in various applications, such as biomedical engineering and optical-based instruments (Lussier et al., 2020; Shin et al., 2020). Three machine learning algorithms, including logistic regression (LR), random forest (RF), and support vector machines (SVM) were applied for classifying features. The accuracy, precision, recall, and F1-score were used as evaluation metrics for classification performance. The overall schematic representation of the experiments and detection principle has been illustrated in Figure 1. The results demonstrated that the proposed technique is effective for the rapid detection and classification of microparticles. The proposed research aimed to shorten detection time, reduce detection costs, and simplify operation methods for classifying microparticles. The detection process did not require expensive equipment and complex operations to perform non-contact, non-invasive, and rapid detection of samples, which had great potential for optical and clinical applications.

## 2 Materials and methods

### 2.1 Micro-particles characteristics

**FIGURE 2:**
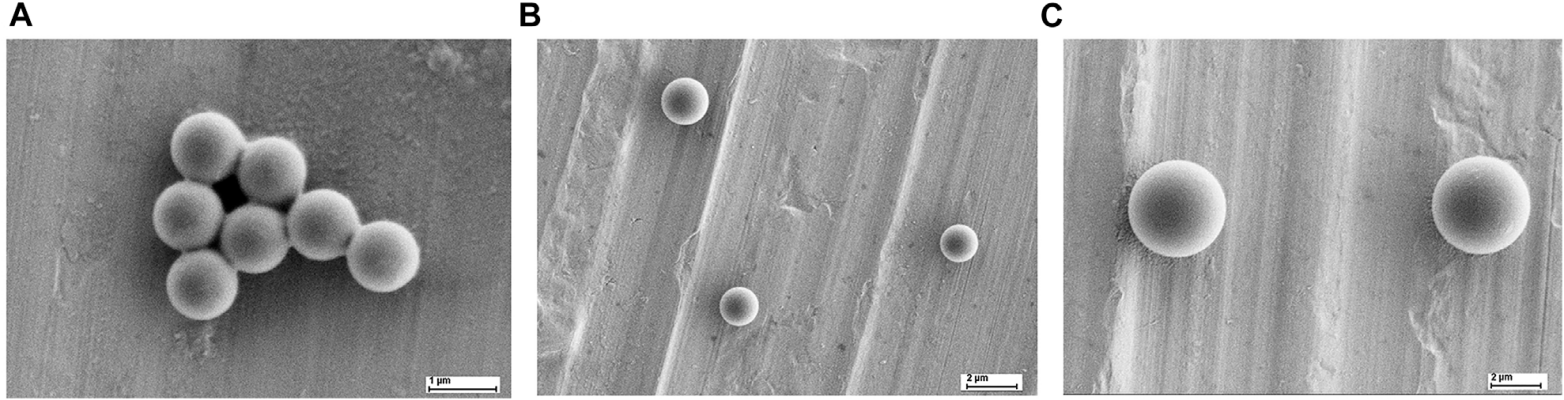
Images of three particles scanning under electron microscope **(A)** 1 µm **(B)** 2 µm and **(C)** 4 µm.

Lumisphere polystyrene fluorescent microspheres from Tianjin Bessler Chromatography Technology Development Center (Tianjin, China) were used with a concentration of 25 mg/ml. The microscopic images of the microparticles with three different sizes were taken by scanning electron microscopy (SEM), as shown in Figure 2. The testing samples of different concentrations were prepared using series dilution to find the optimum concentration for acquiring data. The optimum concentration of the testing sample was obtained by getting the highest number of peaks/variations in the acquired waveform of respective sample. The testing samples with concentrations of 0.0125, 0.025, 0.05, 0.075, 0.1, and 0.125 mg/ml were mixed with DI water to acquire the optimal concentration. The solution was vortexed and centrifuged at 1,000 rpm for 10 min. The residue was removed from the sample to remove unwanted impurities. Then 1 ml of DI water was mixed with the supernatant to prepare the final sample for the experiment. All the experiments were performed at a room temperature (25–28°C).

### 2.2 MDLS prototype

Mie scattering theory describes the elastic scattering of light when the wavelength of the incident light is similar or smaller than the diameter size of spherical particles. Mie theory has been widely applied to laser diffraction analysis to detect particle size effects (Altug et al., 2022). MDLS combines the angular information of Mie scattering with dynamic light scattering to measure particle size. MDLS shows that the intensity of the scattered light fluctuates in a particular direction with time because the tiny particles in solution are in Brownian motion, and the distance between each scattered particle constantly changes with time. MDLS is a common method for detecting particle size and has been used in many medical detection devices (Guo et al., 2018). Optical methods were widely used as an important research tool for particle classification and qualitative detection (Fan et al., 2014; Olson et al., 2015).

**FIGURE 3:**
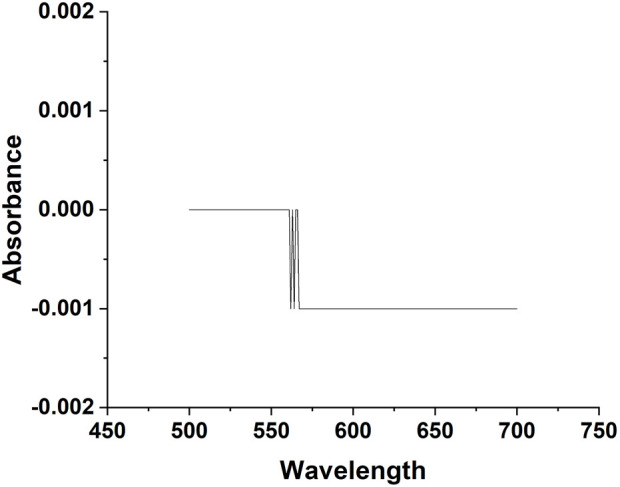
Testing the absorbance of glass flasks used for sample detection from 500 to 700 nm.

The prototype contains three main parts: 1) a laser source, 2) an assembly of the photosensitive sensor, and 3) a data acquisition system (Hussain et al., 2020a). The designed prototype and the laser hardware were assembled by the Nanjing Institute of Advanced Laser Technology, Chinese Academy of Sciences (Nanjing, China). The laser source of the device has a wavelength of 660 nm at a rated power of 150 mW. The power rating of the laser source was measured by PM320E and S130C instruments developed by Thorlabs (New Jersey, United States). When the laser passes through the sample, the scattering light was detected by the high-speed silicon photodiodes FDS100 (wavelength ranges from 350 to 1,100 nm with rise time of 10 ns) manufactured by Thorlabs (New Jersey, United States). The AD8675 manufactured by Analog Devices (Massachusetts, United States) operational amplifier was used in the system to amplify the weak signal. A small flask made of round bottom silicon was used in the experiment (Celo Measure & Control Technology Co., Hefei). UV-1800 Spectrophotometer (SHIMADZU, Japan) measured the absorbance of the glass (Figure 3), which was approximately zero between 500–700 nm wavelength.

The original prototype was designed with 32 photoelectric sensors for signal acquisition (Hussain et al., 2020b). However, the initial number of 32 sensors consumed more computational power. The redundant data acquisition channels did not provide enough information, significantly deteriorating the classification results. The intensity of scattering light depends on the shape, size, and characteristics of the particles based on the Mie scattering theory. Particles of different sizes exhibited different scattered light patterns. The maximum scattering light intensity occurs in the incoming light’s forward direction (Lock and Laven, 2011). Therefore, the number of sensors was tested and reduced from 32 to 12.

**FIGURE 4:**
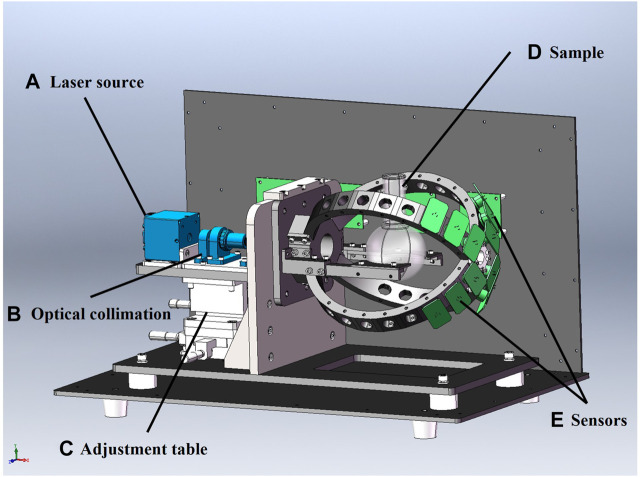
Prototype of the optical system designed by Soildworks 2020. **(A)** Laser source: Laser source with wavelength of 660 nm and power rating of 150 mW laser sources was used to emit laser beam; **(B)** Optical collimation: The optimized collimation system was used to ensure that the scattering light signal from the micro-particles was fully transmitted to the detector unit in real time. **(C)** Adjustment table: The 3-axis positioning system was used to adjust the position of the horizontal x and y axis and the vertical z axis so that the laser beam can be focused on the center of the sample. **(D)** Sample: Different concentrations of microparticle samples mixed with DI water was prepared as experimental samples. **(E)** Sensors: A photodiode with wavelength range of 350–1,100 nm and rise time of 10 ns was implemented to convert the light signal to an electrical signal.

The 3D assembly of the developed prototype was designed using Solidworks 2020 software, as shown in Figure 4. The 3-axis positioning table controlled the “XYZ” 3-axial alignment of the light source to ensure that the laser is focused on the center of the sample. The laser beam positioning unit, laser collimator, sample flask and the direction of the incident light to the sample were optimized to acquire maximum signal energy from the forward scattering light. The calibrated system guarantees that no interference from external factors appears throughout the detection experiments. The signals were collected by NI data acquisition card (PCI-6225). The system showed zero voltage in the dark environment. The data collector maintained the calibrated zero signal waveform when the flask was filled with DI water as an empty sample. The collected data were further processed for features evaluation and data classification using MatlabR 2018a software.

### 2.3 Features extraction and features selection

#### 2.3.1 Features extraction

Signal-to-noise ratio (SNR) gives the ratio between the power of the information signal carried in the acquired signal to the noise signal and measures the quality of the signal. Higher SNR indicates better signal quality and provides valid information for computational analysis (Szkulmowska et al., 2005). The SNR calculated the quality of the signal, where $P_{s}$ denoted the total signal power and $P_{n}$ denoted the noise power, as presented in Eq. 1:

$$\begin{array}{l} SNR=10\mathrm{lg}\frac{P_{s}}{P_{n}} \end{array}$$ (1)

Various time-domain feature extraction algorithms have been developed for signal classification tasks (pahuja et al., 2022; Wang et al., 2022). Rami et al. developed an Electromyography (EMG) signal-based feature extraction method to extract power spectrum features from the non-stationary signal in time domain (Khushaba et al., 2014). The acquired data from the prototype also showed non-stationary behavior. In the feature extraction technique, the MDLS signal is denoted by x [*j*], the length of the signal is represented by *N,* and the sampling frequency is *fs*. The MDLS scattered signal can be expressed over time by a function of X [*k*] after the discrete Fourier transform (DFT). According to Parseval’s theorem, the power contained in the signal is equal to the sum of the powers of the components of the signal from the complete orthogonal set. The Parseval’s theorem is applied to the derivation of the power spectral characteristics:

$$\begin{array}{l} \overset{N-1}{\underset{j=0}{\sum }}{\left|x\left[j\right]\right|}^{2}=\frac{1}{N}\overset{N-1}{\underset{k=0}{\sum }}\left|X\left[k\right]X^{*}\left[k\right]\right|=\overset{N-1}{\underset{K=0}{\sum }}P\left[k\right] \end{array}$$ (2)

The power spectrum characteristic *P* [*k*] is calculated by multiplying $X^{*}\left[k\right]$ and *X* [*k*], where *k* denotes the frequency of the signal. Eq. 2 represents the phase-excluded power spectrum.

According to the symmetry of the Fourier transform, all positive and negative frequencies were included to handle the whole spectrum. The given method used the time domain signal to evaluate the power spectrum features. All odd moments were set to zero by the FT. Therefore, according to the definition of the *nth* order moment m of the power spectral density P [*k*], all odd moments will be calculated as zero, which is defined as:

$$\begin{array}{l} m_{n}=\overset{N-1}{\underset{k=0}{\sum }}k^{n}P\left[k\right] \end{array}$$ (3)

Based on Eq. 2, Parseval’s theorem can be used when *n* = 0. According to the time-differentiation property of the FT, when *n* ≠ 0, the *n* ^*th*^ order derivative of the discrete time signal equals the spectrum multiplied by the nth power of *k* as follows:

$$\begin{array}{l} F\left[\Delta^{n}x\left[j\right]\right]=k^{n}X\left[k\right] \end{array}$$ (4)

Therefore, the number of extracted features is defined by the following properties:

##### 2.3.1.1 Zero-order moments

The zero-order moment represented the total power in the frequency domain:

$$m_{0}\begin{array}{l} =\sqrt{\overset{N-1}{\underset{j=0}{\sum }}x{\left[j\right]}^{2}} \end{array}$$ (5)

##### 2.3.1.2 Second-order moments and fourth-order moments

The second-order moment indicates the magnitude of the fluctuation of the power spectrum corresponding to the mean value. In the power spectrum characteristics, the power spectrum with $k^{2}P\left[X\right]$ is correlated with spectrum related to $kX\left[k\right]$ , and represented by Eq. 2:

$$m_{2}\begin{array}{l} =\sqrt{\overset{N-1}{\underset{k=0}{\sum }}k^{2}P\left[k\right]}=\sqrt{\frac{1}{N}\overset{N-1}{\underset{j=0}{\sum }}\left(\Delta x\left[j^{2}\right]\right)} \end{array}$$ (6)

Similarly, the fourth-order moments can be expressed by the following equation:

$$m_{4}\begin{array}{l} =\sqrt{\overset{N-1}{\underset{k=0}{\sum }}k^{4}P\left[k\right]}=\sqrt{\frac{1}{N}\overset{N-1}{\underset{j=0}{\sum }}{\left(\Delta^{2}x\left[j\right]\right)}^{2}} \end{array}$$ (7)

The features were normalized ( $m_{0}$ , $m_{2}$ and $m_{4}$ ) to reduce the effect of noise on the features, where λ was routinely set to 0.1.

$$\begin{array}{l} m_{0}=\frac{m_{0}^{\lambda }}{\lambda },m_{2}=\frac{m_{2}^{\lambda }}{\lambda },m_{4}=\frac{m_{4}^{\lambda }}{\lambda } \end{array}$$ (8)

Thus, the first three extracted features $f_{1},f_{2},f_{3}$ are defined as:

$$\begin{array}{l} f_{1}=\log\left(m_{0}\right) \end{array}$$ (9)

$$\begin{array}{l} f_{2}=\log\left(m_{0}-m_{2}\right) \end{array}$$ (10)

$$\begin{array}{l} f_{3}=\log\left(m_{0}-m_{4}\right) \end{array}$$ (11)

##### 2.3.1.3 Sparsity

Sparsity defines the energy contained in a vector which is represented by:

$$\begin{array}{l} f_{4}=\log\left(\frac{m_{0}}{\sqrt{m_{0}-m_{2}}\sqrt{m_{0}-m_{4}}}\right) \end{array}$$ (12)

##### 2.3.1.4 Wavelength ratio

The waveform length characteristic defines the sum of the absolute values of the second and fourth-order derivatives of the signal:

$$\begin{array}{l} f_{5}=\log\left(\frac{\sum_{j=0}^{N-1}\left|\Delta^{2}x\right|}{\sum_{j=0}^{N-1}\left|\Delta^{4}x\right|}\right) \end{array}$$ (13)

However, the sampling frequency of the data acquisition was set to 1 kHz for the 12 channels. The five features were collected from each channel to form the feature vector $f=\left[f_{1},f_{2},f_{3},f_{4},f_{5}\right]$ . A total of 60 features were extracted from 12 channels, and the feature filtering algorithms were applied to filter the feature set.

#### 2.3.2 Features selection method

The dimensionality of features affects the classification results, and irrelevant features may degrade the performance of the classifier (Li et al., 2017). Feature selection methods optimize the feature set and remove low relevance and redundant features. Feature selection methods are useful for removing irrelevant features affecting the training model and improving the classification accuracy of the model (Khaire and Dhanalakshmi, 2022). The PCA and RFE were used as dimensionality reduction algorithms.

##### 2.3.2.1 PCA

PCA is a commonly used algorithm for data dimensionality reduction. PCA can project each data point onto only the first few principal components to obtain low-dimensional data while preserving as much variation as possible. In PCA, high-dimensional features are determined based on the eigenvectors and eigenvalues of the covariance matrix for K principal components and mapped to the K-dimensional space (K < N), where K-dimensional features are associated as new eigenvectors based on the magnitude of the eigenvalues (Yang et al., 2004).

##### 2.3.2.2 RFE

Removing several features at a time often negatively impacts classifier performance, while using a small subset may yield better results. Therefore, RFE was introduced to overcome the drawback (Guyon et al., 2002). REF is a feature selection method with good generalization performance based on backward search of the model as features were removed in each iteration by using the feature importance metric within the model as a metric. RFE searches for a subset of features by starting with all features in the training dataset and successfully removing features until the desired numbers are obtained.

### 2.4 Machine learning

Machine learning represents a class of algorithms for data classification. With the development of artificial intelligence, machine learning technology has been widely applied in various fields, especially in biomedical engineering and life science (Goodswen et al., 2021). Among different machine learning algorithms, LR is a probabilistic non-linear regression with applications in prediction and discrimination. RF is an integrated learning model that contains multiple decision trees, and the output category is determined by the plurality of the categories output from the individual trees. SVM is a typical supervised learning classification algorithm in machine learning, which classifies data by finding the maximum interval in the feature space (Ghannam and Techtmann, 2021).

**FIGURE 5:**
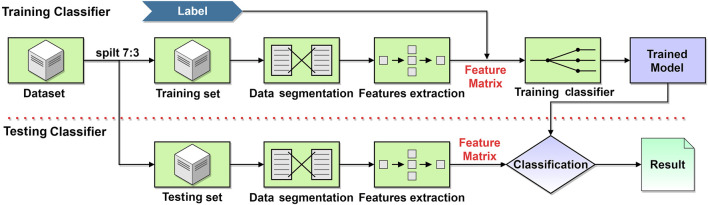
Flowchart of the microparticles classification based on machine learning.

After acquiring the experimental data, the machine learning classification algorithms were applied based on Sklearn 0.19.0 package (Intel Core i7-10700 CPU processor, 2.90 Ghz, 32 GB RAM, RXT 3070). The SVM classifier acquired the highest classification accuracy compared with other classifiers. The machine learning classification process is presented in Figure 5, where the data set was divided into a training set and a test set in the ratio of 7:3. After denoising, the data was segmented by adding a sliding window length of 250 ms and slid over the data signal with an increment of 150 ms. The power spectrum features were extracted for the signals in each window. The features extracted from three particle sizes of 1, 2, and 4 µm were labeled before training the machine learning classifier.

### 2.5 Performing analysis and features contribution

#### 2.5.1 Performing analysis

Model evaluation metrics were applied to select a model with high generalization ability for machine learning classification tasks. Models with high generalization ability tend to adapt the unknown samples. Cross-validation is used to assess machine learning classifiers on the training set for checking their performance. In 5-fold cross-validation, the dataset is divided into 5 subsets that are not utilized in training the classifier. The 5-subsets were used for testing the model to evaluate the classifier (Bergmeir and Benitez, 2012; Wong and Yeh, 2020). The performance was assessed based on the average value generated from each result of the subset. In this research, the 5-fold cross-validation was performed to optimize the parameters of the classifier. The generalization performance of the model was tested on the test set.

The confusion matrix was plotted for subsequent computation of performance evaluation metrics. The confusion matrix is a class of tables used to visualize the classification results and evaluate classification performance. The values from the confusion matrix were represented by True Positive (*TP*), True Negative (*TN*), False Positive (*FP*), and False Negative (*FN*). The given characteristics were applied to evaluate the performance metrics, in which *TP* and *TN* denote the results of particle size that had been correctly classified, while *FP* and *FN* represent the results of particle size data classified incorrectly by the classifier. These parameters constituted the confusion matrix were used to evaluate the performance metrics, including precision, accuracy, recall, and F1 score. Precision represents the percentage of correctly predicted results to total outcomes. Recall is the probability that the predicted positive samples were positive samples. The F1 score was obtained from the weighted average of precision and recall.

The above metrics were used to evaluate the performance of the classifier and defined respectively:

$$\begin{array}{l} Accuracy=\frac{TP+TN}{TP+TN+FP+FN} \end{array}$$ (14)

$$\begin{array}{l} Precision=\frac{TP}{TP+FP} \end{array}$$ (15)

$$\begin{array}{l} Recall=\frac{TP}{TP+FN} \end{array}$$ (16)

$$\begin{array}{l} F_{1}score=\frac{2\times TP}{2\times TP+FP+FN} \end{array}$$ (17)

#### 2.5.2 Features contribution

Shapley value is a solution concept that involves the equitable distribution of benefits and costs to several actors working jointly in the game theory (Ribeiro et al., 2016; Lundberg and Lee, 2017). Shapley values are mainly applied to situations where the contribution of each actor is not equal but cooperates to obtain a benefit or reward. Shapley values have been widely used in artificial intelligence to provide good interpretability for machine learning and deep learning black box models (Rozemberczki et al., 2022). The proposed method can attribute the output value of the model to each Shapley value in the dataset at each sample level. Shapley values provide a natural way to calculate which features contribute to predictions, interpreting a model trained on a set of features as a coalition of players’ value functions. The Shapley value explains the degree of contribution of each feature to the outcome.

## 3 Results

### 3.1 Prototype design

**FIGURE 6:**
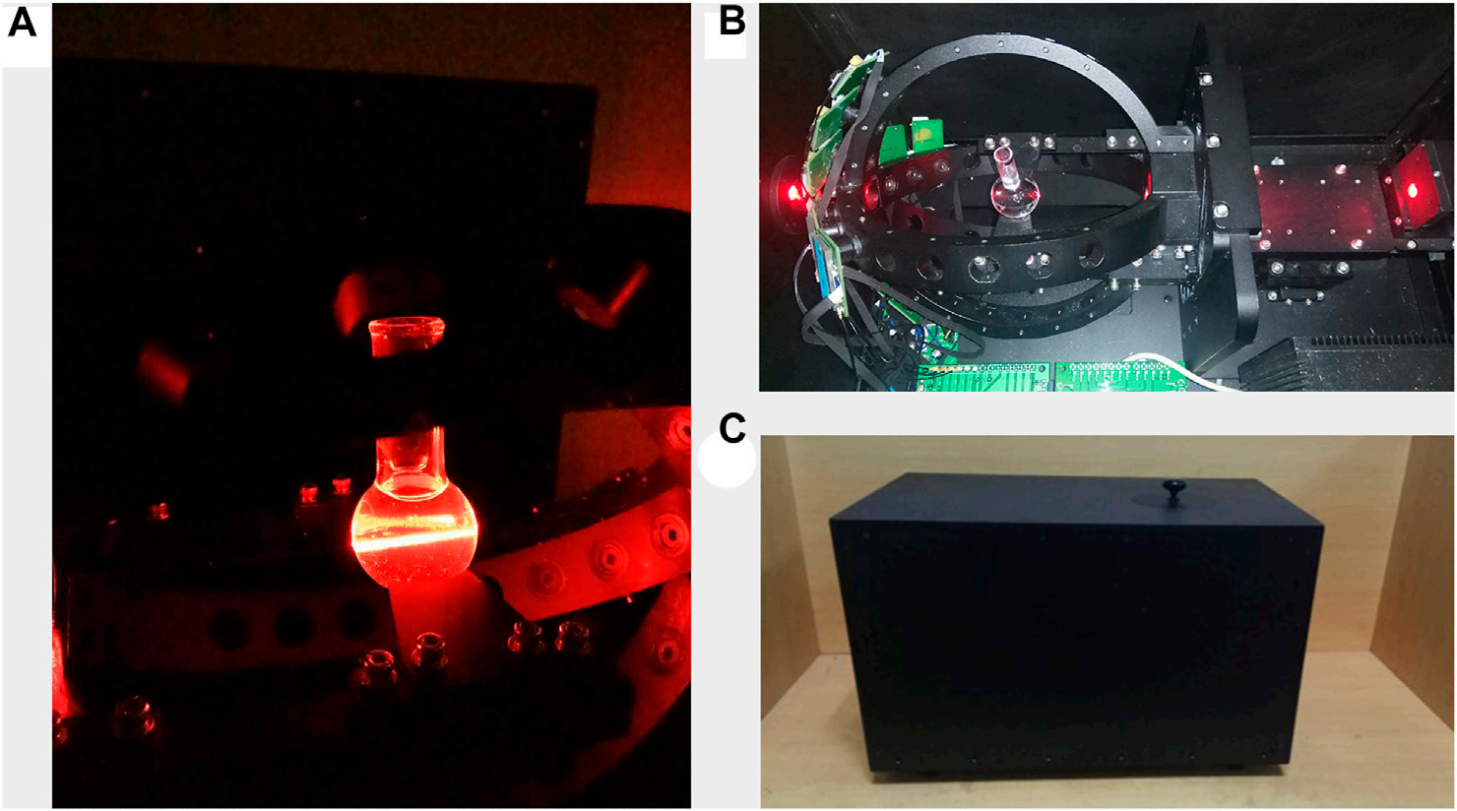
**(A)** Internal status of the prototype during the operation; **(B)** photodetectors are distributed in the backward direction; and **(C)** an external cover of the system is used to avoid external light (dimension: 50 cm × 30 cm × 30 cm).

The prototype was successfully developed with a dimension of 50 cm × 30 cm × 30 cm. The testing sample was placed in the chamber of the prototype, as shown in Figure 6A. The sample was placed to focus the laser light on the center of the flask, as shown in Figure 6B. The particles that randomly moved in the flask scattered the detection beam while the sensor received the scattered signal in real-time. The pattern of scattering light was affected by the size and shape of the microparticles (Hussain et al., 2019). A black box was used to avoid external interference, improving the quality of the acquired signal, as shown in Figure 6C.

### 3.2 Optimized condition experiments

**FIGURE 7:**
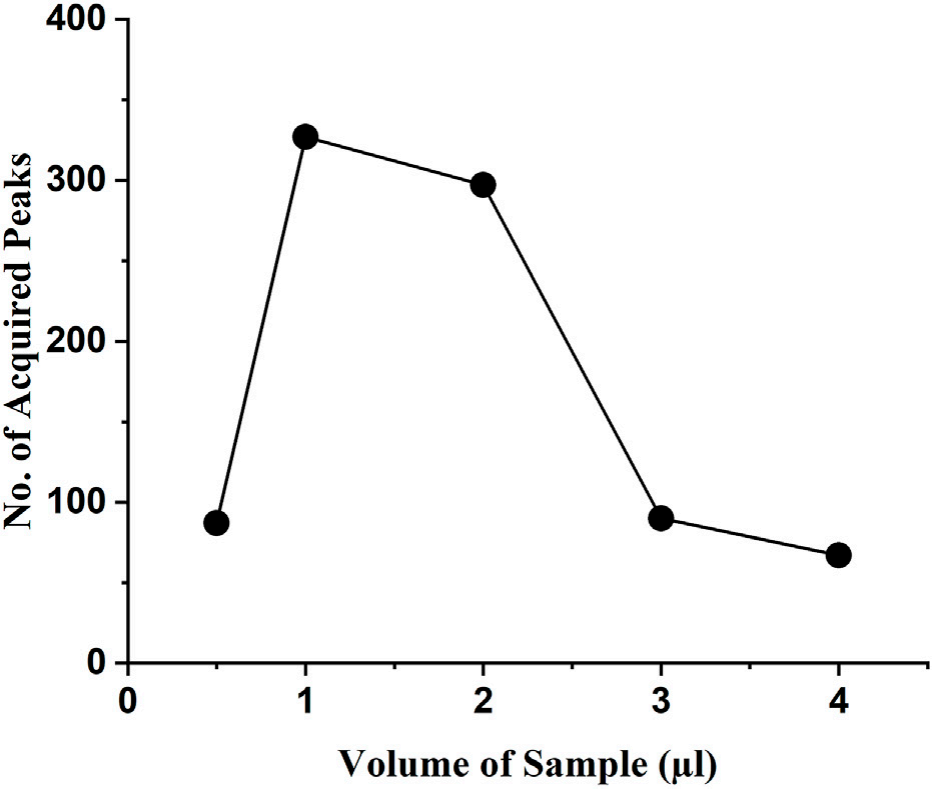
Average peak values were obtained using samples with different concentrations.

The testing samples with concentrations of 0.0125, 0.025, 0.05, 0.075, 0.1, and 0.125 mg/ml were mixed with DI water to acquire the optimal concentration. Each of the experiments was performed for 30 min. Samples with different concentrations were tested to obtain the average number of peaks (Figure 7). The scattering of the light was weak when the concentration was too low, so the number of detected peaks was less. At higher concentrations, the number of detected characteristic peaks was also too low due to particle-to-particle interaction. Overall, the experimental results showed that a higher number of peaks in the waveform were generated at a sample concentration of 0.025 mg/ml. The classification outcomes showed incorrect results for samples with concentrations below 0.025 mg/ml. Therefore, the sample concentration of 0.025 mg/ml is considered as the detection limit.

### 3.3 Signal testing

**FIGURE 8:**
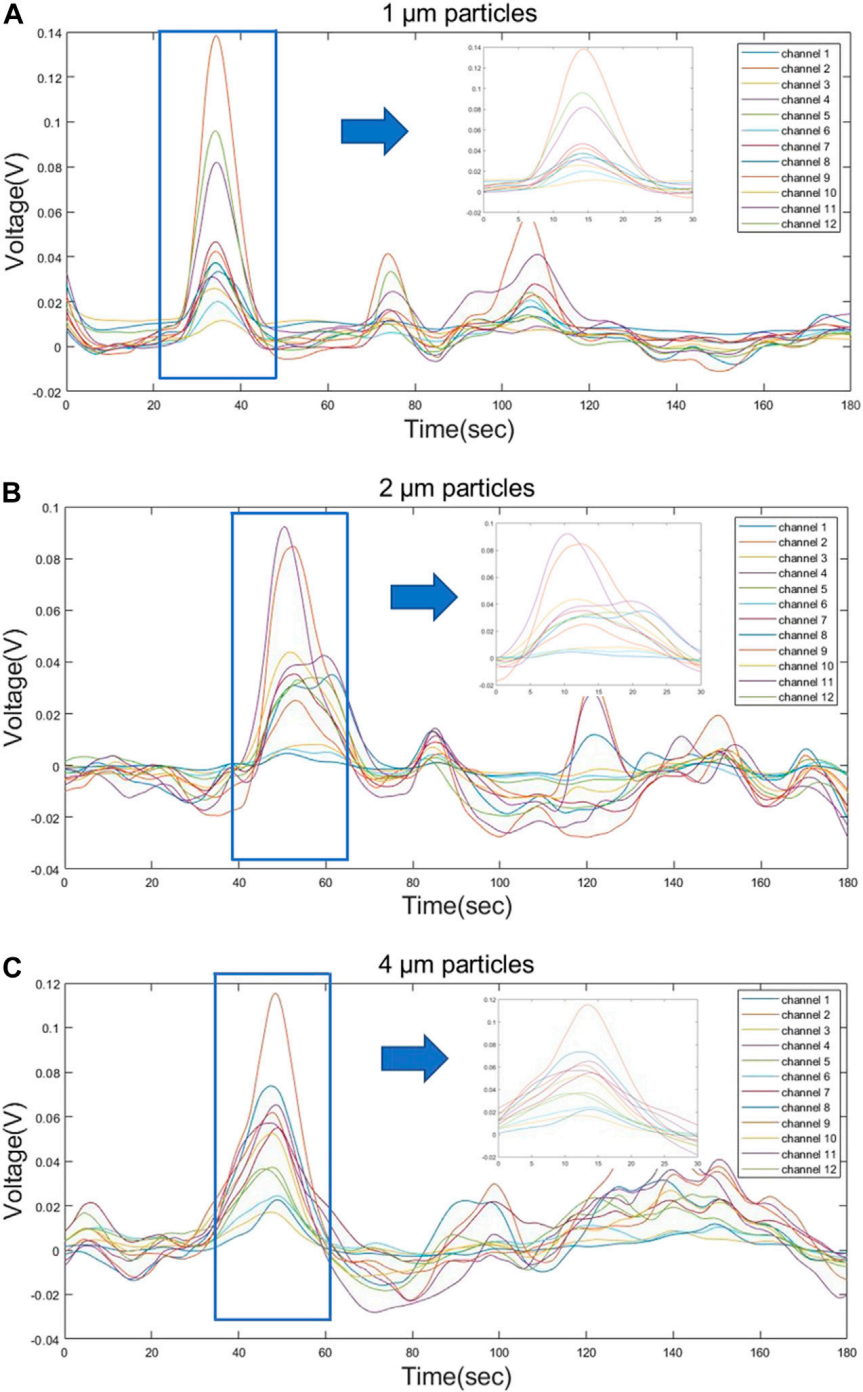
Partial waveform signals from three different sizes of particles **(A)** 1 µm **(B)** 2 µm and **(C)** 4 µm appeared during the 180 s of detection, during which the particles passing through the beam showed a significant voltage signal on the sensor.

The prepared samples with an optimum concentration were used to detect the MDLS. The time-domain light scattering signals from three different particles were obtained for 30 min. The SNR of the signal increased from 2.98 to 6.698 after applying the second order Butterworth filter to the acquired raw data. Figures 8A–C shows the test signal obtained from 12 channels for three particles with time duration of 3 min. An output signal shows significant variations in the peak values obtained from different particles sizes. The peak values represent the time when the particles pass through the detection beam. The signal test significantly revealed the similarity of the particle signals of the three different particle sizes in terms of peak features and combinations of detection channels, so the feature extraction and machine learning approach can significantly improve the effectiveness of the classification task.

### 3.4 Features reduction and classification algorithms

**FIGURE 9:**
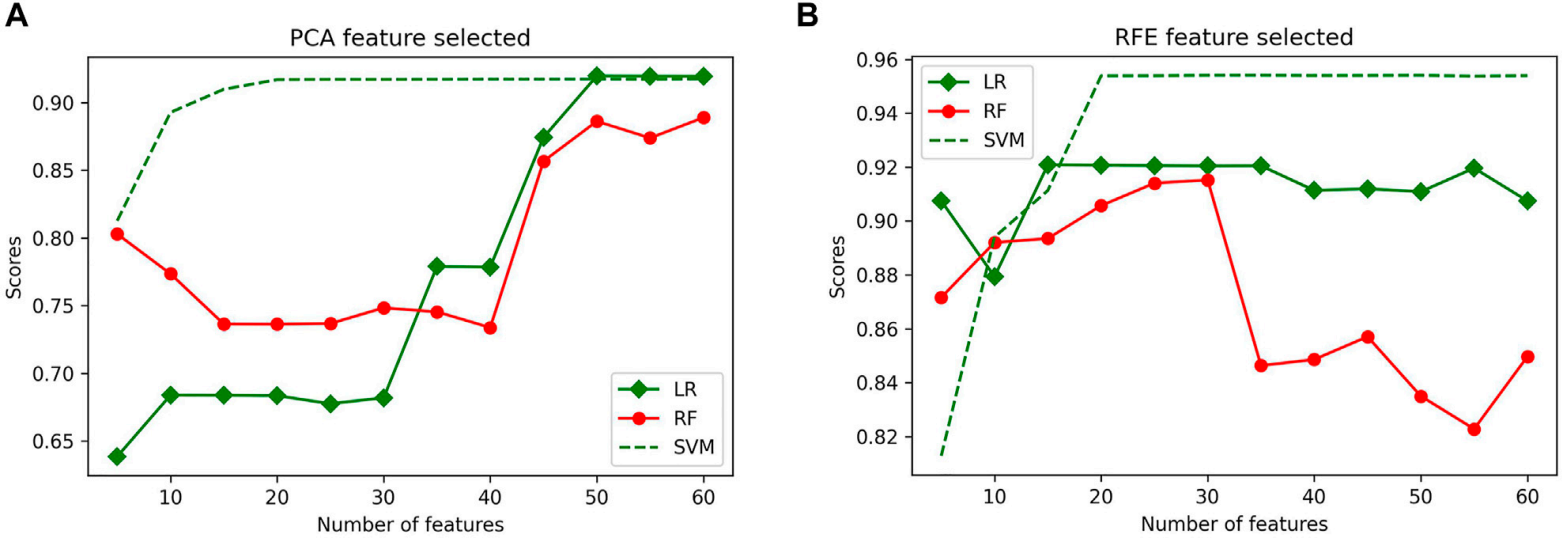
Average classification scores obtained from LR, RF and SVM classifiers using **(A)** PCA and **(B)** RFE for feature selection after 5-fold cross-validation.

PCA and RFE were used as feature selection algorithms, and then 5-fold cross-validation was applied to the features. Before training and testing the classifiers, the features matrix were labeled as 1 µm (class 0 label), 2 µm (class 1 label), and 4 µm (class 2 label). The number of features varied from 5 to 60, with an increment of 5 in each iteration. A 5-fold cross-validation evaluated the results of each feature selection. The highest accuracies of LR, RF, and SVM classifiers using 50 selected features with PCA were 91.97%, 88.89%, and 91.74%, respectively (Figure 9A). Similarly, the highest accuracies of LR, RF classifiers were 92.08%, 91.52%, using 30 selected features, the highest accuracy of SVM classifier were 95.38% using 50 selected features with RFE, (Figure 9B). Overall, the classification results using RFE were more accurate than PCA. The RFE feature selection method selected a subset of 50 features to obtain the highest classification accuracy.

**FIGURE 10:**
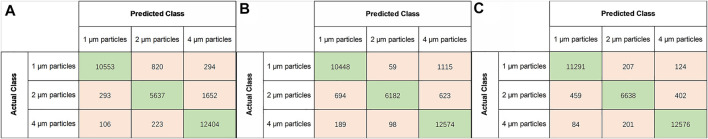
Confusion matrix of three different classifiers **(A)** RFE-LR **(B)** RFE-RF **(C)** RFE-SVM on testing set.

The confusion matrix was plotted from the outcomes to evaluate the performance of the three classifiers. The row of the confusion matrix represented the actual sample classes, and the column represented the predicted sample classes (Figure 10). The green boxes in the diagonal line represented the outcomes that were correctly classified. The remaining feature data points represented the incorrectly classified values. The identification accuracy, precision, recall, and F1 score of the testing dataset were evaluated using Eqs 14–17, and the results are presented in Table 1. Overall, the SVM(kernel = “linear”, decision_function_shape = “ovr”,C = 100, gamma = 0.0001, probability = True) classifier achieved higher classification accuracy than LR and RF classifiers.

**TABLE 1:** Four evaluation metrics of different classifiers on test dataset.

Model evaluation metrics	LR	RF	SVM
Accuracy (%)	92.26	91.31	95.38
Precision	0.9238	0.9170	0.9536
Recall	0.9226	0.9131	0.9538
F1 score	0.9215	0.9125	0.9634

### 3.5 Features contributions

**FIGURE 11:**
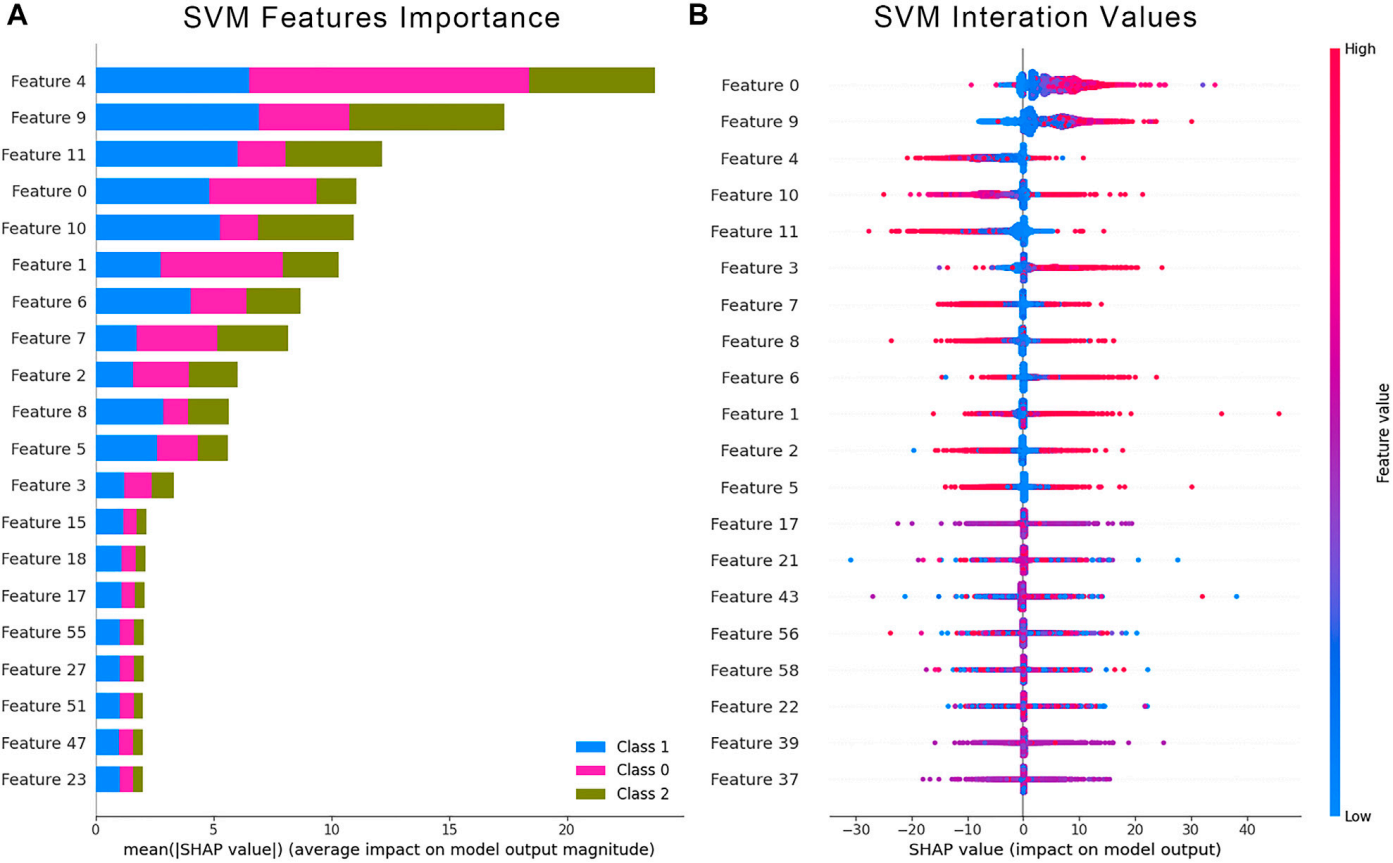
Quantification of feature impact on prediction through analysis of Shapley additive explanations (SHAP) values of the 20 most impactful features. **(A)** Averaged Shapley values of features with higher contribution indicate an average effect of these features on the output amplitude. **(B)** The scatter plot represents the distribution of Shapley values and the effect on the model output of all test samples.

The Shapley values were used to validate whether the 50 power spectrum features subset influenced the prediction results. We computed the Shapely values that were contributed from the power spectral features of the SVM classification model. The average Shapely values from each feature and the corresponding Shapley values for each data point were calculated and counted. The bar chart on the left (Figure 11A) showed the average Shapley values for the three classifiers, indicating each feature’s average contribution to the final output. The scatter plot on the right (Figure 11B) demonstrated the distribution of Shapley values and their contribution to the model output structure. The color of each point represented the intensity of the feature value. Blue data points indicated low features, while red represented the opposite. The results showed a significant contribution of the first 12 features to the results, consistent with the proposed channel arrangement for the acquisition system. The first 12 features that corresponded to the zero-order moment features of the 12 channels played a crucial role in the contribution of the classification results.

## 4 Discussion

The objective of this paper is to develop a new system and to verify the ability of the proposed technique for the identification of different particle sizes. With the continuous development of point-of-care testing (POCT) technology, detection techniques are gradually evolving with high accuracy and simplicity. The developed system can identify the size of microparticles with high accuracy in a short time. The given analysis was conducted by collecting a large amount of data based on the scattering light from particle samples of different sizes (1, 2, and 4 μm). The time-domain features were obtained from the acquired data, and the features were reduced the number of features to acquire higher classification accuracy. The RFE feature selection method selected a subset of 50 features, and gave best results compared with PCA features selection method. The selected features were trained using machine learning to automate the detection procedure. The logistic regression classifier gave classification accuracies of 95.30%, 92.38%, 89.17% for particle sizes of 1, 2 , and 4 μm, respectively. The random forest classifier gave classification accuracies of 92.59%, 96.71%, 76.62% for particle sizes of 1, 2, and 4 μm, respectively. The SVM classifier showed higher identification accuracy with prominent classification parameters. The SVM classifier gave the highest classification accuracies of 94.41%, 94.20%, 96.12% for particle sizes of 1, 2, and 4 μm, respectively. The trained SVM classifier gives an average classification accuracy of 95.38%. The detection limit of the given method is 0.025 mg/ml. The contribution and effect of each feature to the results were analyzed by features selection methods. Selected features identified by the RFE feature filter have shown superior classification results. The Shapley values of these features described a significant contribution to the results. In summary, the developed system based on MDLS and machine learning can quickly and accurately detect microparticles. Furthermore, the prototype was highly integrated, and the developed method did not require lengthy sample preparation. The given technique requires further validation for practical applications that can be applied to detect microbial particles within the range of 1–4 µm.

## Data Availability

The raw data supporting the conclusion of this article will be made available by the authors, without undue reservation.
